# A Growing Mandate in American Medicine: Teaching Our Internal Medicine Residents About Healthcare Administration and Leadership

**DOI:** 10.7759/cureus.49487

**Published:** 2023-11-27

**Authors:** Carolina Borz-Baba, Anupama Paranandi, Shawnette Alston

**Affiliations:** 1 Internal Medicine, Saint Mary's Hospital, Waterbury, USA; 2 Family, Population, and Preventive Medicine, Stony Brook University, New York, USA

**Keywords:** curriculum, education, internal medicine residency, leadership, healthcare administration

## Abstract

Leadership training during residency is essential for the successful development of contemporary physicians. Creating a curriculum for healthcare leadership and administration for internal medicine residents is particularly challenging due to the heterogeneity of leadership curricula across programs, the emphasis on individual advancement rather than collective leadership, and the scarcity of published research on the topic. A healthcare administration and leadership rotation for medical residents is a valuable experience that emphasizes the importance of contextualizing education on leadership and building relationships to achieve organizational goals.

## Editorial

Incongruent conflations

There is an expanding interest in leadership development programs among physicians, and formal training is currently available in 65% of academic health centers in the United States of America [1]. The enthusiasm for physician leadership training among residents is increasing [2], and the necessity to educate them on such a topic is evident [3], as residents are expected to participate in multidisciplinary rounds, lead a team, be active advocates of patient safety, and share the responsibilities of educating junior residents and students. The most recent Accreditation Council of Graduate Medical Education (ACGME) 2.0 Milestones [4] highlight the importance of advancing resident performance in leadership in most competency areas; thus, early exposure to healthcare administration and leadership is paramount to the contemporary internal medicine (IM) trainee.

It is essential to clarify the existing distinction between healthcare administration and leadership. Although the terms are commonly used interchangeably, they are two separate concepts. While leadership refers to an individual’s attribute to guide others toward a common goal, healthcare administration involves the establishment of objectives for effective operations and requires knowledge of policies and procedures. It is unequivocal that a healthcare leader must have expertise in healthcare administration, but it is critical for a healthcare administrator to possess leadership skills. By preserving the difference between the two constructs, we emphasize not their separation but their interdependence.

Healthcare administration and leadership elective at a community hospital

To advance our IM residents’ knowledge in healthcare administration and leadership, our program created the administrative elective rotation in 2016 and further refined it as the administrative and leadership elective in 2023. The elective rotation is a four-week rotation available to postgraduate year two (PGY2) and postgraduate year three (PGY3) residents who are interested in becoming physician leaders.

The curriculum aims to expand residents’ knowledge, skills, and professional attitudes toward healthcare administration and leadership. Throughout the academic year, our IM residents attend noon conferences and grand rounds on a variety of relevant topics including leadership development, healthcare costs and disparities, coding, patient safety, and patient experience, to name a few. They also participate in team-building exercises. In addition, the trainees who participate in the elective are required to work directly with the chief medical officer (CMO) and attend a series of hospital-based meetings on patient safety, patient experience, and hospital quality metrics. In addition, they attend executive conferences, multidisciplinary rounds (designed to assist with patient discharge in real-time), and leadership development sessions.

**Figure 1:**
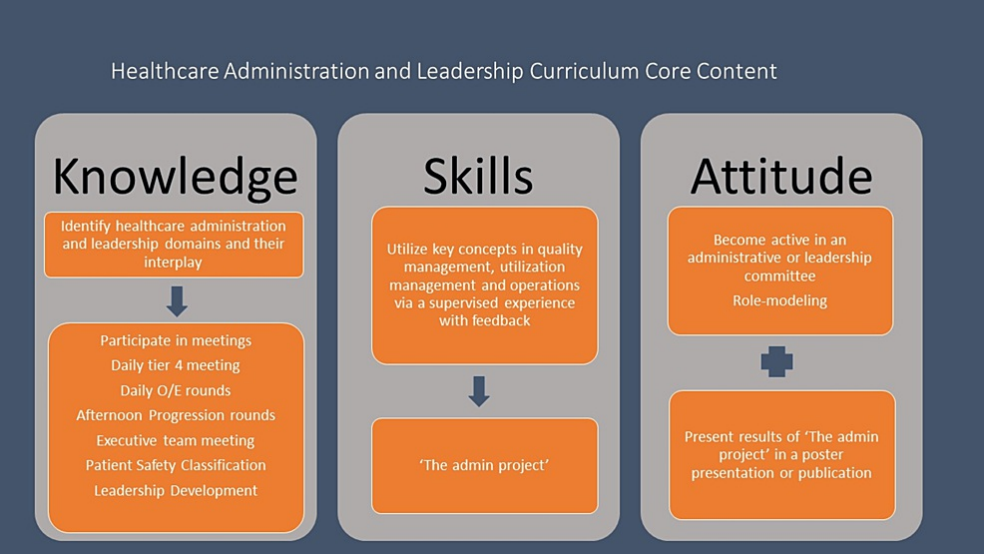
Healthcare administration and leadership curriculum

To advance their skills and their professional attitude, trainees must complete an administrative project and engage in leadership training under the direct guidance of the CMO. The administrative project is a quality improvement (QI) project conducted by the resident and supervised by the CMO. The project is developed based on the I-SMART criteria (I-important, S-specific, M-measurable, A-accountability, R-realistic, T-timeline). The results of their project are presented at an educational session, typically a noon conference or a scientific conference, after the elective is completed. The evaluation of the residents is based on a cumulative assessment of their attendance at the required meetings and direct CMO observation of their interactions with interdisciplinary teams, including QI representatives, and other executives (e.g., the chief nursing officer). Their performance on the administrative project is evaluated separately. The core content of the curriculum is depicted in Figure 1.

Compare and contrast

Existing evidence about the efficacy of leadership programs for graduate medical trainees [5] is sparse and conflicting. There is a significant misconception among trainees who appreciate that leadership development is primarily about enhancing individual knowledge and skills in the hierarchical and archaic model of leadership [2] at the level of a microsystem. The existent leadership programs have not adopted contemporary views of leadership as a collective practice, and there is little evidence to support their efficacy. Imperfect as they are, existing sources represent a fountain of wisdom available for programs interested in developing leadership experiences.

With regard to establishing a curriculum for healthcare administration and leadership for IM residents, we noted some challenges.

The first is the absence of quality evidence to define the competencies and structure required by a formal curriculum [2]. The Healthcare Leadership Alliance [6] and the Academy of Medical Royal Colleges [7] endorse a comprehensive but equally extensive leadership competency framework that exemplifies the complexity of a physician-leadership role. To our knowledge, there is no healthcare administration curriculum for IM residents, but when used interchangeably with leadership, we can appreciate the scarcity of literature and the absence of a shared curricular domain. Some IM leadership programs focused on leadership styles, emotional intelligence, and leading clinical teams [8], while others were more extensive, including training on professionalism, communication, and conflict management [9].

It seems inherent that the resident will acquire knowledge of effective operations, policies, and procedures formulated in the administrative space and that should occur concurrently with leadership training. Residents’ skills in quality, utilization management, and operations and their interconnection become particularly important in small hospitals where trainees may have a closer work relationship with case management, quality, and the executive team. From this derives the hypothesis that by retaining the accent on healthcare administration, the experience promotes the acquisition of the skills required for a shared model for making decisions and collective leadership at the microsystem level.

The second challenge is choosing the setting and the duration. Hospital-based programs [2,10] appear feasible due to the immediate availability of the stakeholders. Approximately 13% of existing training in leadership are hospital-based electives [5], like ours. Most programs (45%) extend to ≥1 year [5]. A longitudinal program covering training from PGY1 to PGY3 or PGY4 seems adequate to progressively build knowledge and skills, particularly in a large university center, but it might not be compatible with community-based hospitals with minimal resources and without access to a national panel of experts in leadership styles, emotional intelligence, and diversity.

In terms of the educational methods, the trainees’ perception is that leadership development needs to be clinically integrated and based on direct interaction with the executive team [2]. Lectures to cover leadership theory, case-based simulations, and workshops are employed as necessary, but the experiential model and project-based approach are essential to apply the concepts studied.

An elective rotation is an excellent opportunity for second- and third-year residents who are interested in an administrative-leadership career to consolidate fundamental concepts learned in the direct work environment via multidisciplinary meetings, incident reports, patient experience reports, and informal educational sessions such as grand rounds and noon conferences. A one-on-one interaction with a physician executive is critical for the trainees to enhance their leadership skills.

An active learning design, including an administrative or leadership project, allows a resident to personalize the rotation and mature their aptitudes. Finally, by mandating that the project be shared with other residents and faculty in an educational session, poster competition, or publication, the trainees take on the role of the educator, share their experiences, and promote their vision.

The key message

A four-week administrative and leadership elective rotation for IM residents at a community-based hospital is a feasible alternative to longitudinal experiences. Refining the experience as an administrative and leadership experience gives the learner the perspective of relational leadership and primes them to engage with a collective leadership philosophy in the future.
